# Characterization and Effects of Fiber Pull-Outs in Hole Quality of Carbon Fiber Reinforced Plastics Composite

**DOI:** 10.3390/ma9100828

**Published:** 2016-10-13

**Authors:** Sina Alizadeh Ashrafi, Peter W. Miller, Kevin M. Wandro, Dave Kim

**Affiliations:** 1 Tube Specialties Company, Troutdale, OR 97060, USA; sashrafi@tubespecialties.com; 2 School of Engineering and Computer Science, Washington State University, Vancouver, WA 98686, USA; peter.w.miller@gmail.com (P.W.M.); kevin_wandro@wsu.edu (K.M.W.)

**Keywords:** carbon fiber reinforced plastic (CFRP), drilling, fiber pull-out, hole quality

## Abstract

Hole quality plays a crucial role in the production of close-tolerance holes utilized in aircraft assembly. Through drilling experiments of carbon fiber-reinforced plastic composites (CFRP), this study investigates the impact of varying drilling feed and speed conditions on fiber pull-out geometries and resulting hole quality parameters. For this study, hole quality parameters include hole size variance, hole roundness, and surface roughness. Fiber pull-out geometries are quantified by using scanning electron microscope (SEM) images of the mechanically-sectioned CFRP-machined holes, to measure pull-out length and depth. Fiber pull-out geometries and the hole quality parameter results are dependent on the drilling feed and spindle speed condition, which determines the forces and undeformed chip thickness during the process. Fiber pull-out geometries influence surface roughness parameters from a surface profilometer, while their effect on other hole quality parameters obtained from a coordinate measuring machine is minimal.

## 1. Introduction

**Figure 1:**
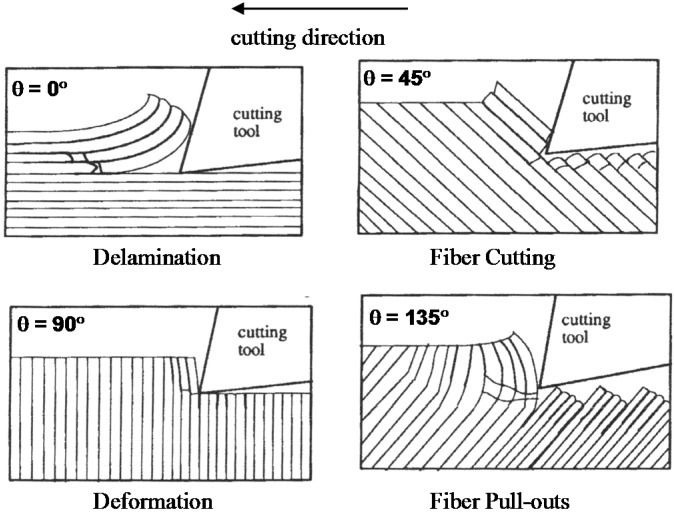
Schematics of CFRP cutting mechanisms depending on fiber orientations [19].

Aircraft manufacturers are putting major effort into producing more fuel-efficient airplanes. This effort will drastically reduce airplane carbon emissions and help improve the environment in a sustainable fashion. Fuel-efficient airplane designs often involve the switch to carbon fiber-reinforced plastic composites (CFRP). The superior advantages of CFRP over conventional metallic materials, include high specific strength, high stiffness, high fatigue resistance, and low thermal expansion. These advantages have given rise to CFRP composite applications in many transportation industries. Mechanical fastening is typically used in aircraft assembly due to its straightforward and lower-risk joining characteristics, high through-the-thickness reinforcement, and ease of repair. Drilling holes is an essential process when using mechanical fasteners to combine two or more CFRP members or CFRP members with metallic members to form structures [1,2,3,4,5,6,7,8]. Close-tolerance CFRP hole generation is very critical when conducting aircraft assembly. Higher precision is often required in aircraft manufacturing compared with other industry sectors. Many studies have been carried out in the last few decades to develop defect-free drilling methods, which include water jets [9], laser cutting [10,11], and the use of vibration-assisted drilling machine tools [12] in addition to the conventional drilling process. However, conventional drilling still remains one of the most widely used processes when generating CFRP holes due to its high productivity and economic reasons [1,2,3,4,5,6,7,8]. The conventional drilling process leads to various CFRP hole quality issues, such as inconsistent hole size, roundness, and surface profile, as well as entry/exit delamination, inter-laminar crack propagation, and fiber pull-out [3,13,14,15,16,17,18]. One of the unique defects observed in CFRP holes is fiber pull-out, which occurs when bundles of carbon fibers are pulled away by fiber-matrix de-bonding and matrix stripping. As shown in Figure 1, fiber pull-outs are commonly observed when the angle between the ply and the cutting direction is close to 135 degrees [18,19,20,21]. These micro level fiber pull-outs can be the origin of fatigue cracking [22,23,24] and failure [25,26] of the CFRP structures while other machining hole defects affect the strength and fatigue life of composites subjected to static and dynamic loading [27,28].

While generating close-tolerance CFRP holes is important, assessing hole quality precisely and economically is also critical for aircraft assembly. The machined CFRP hole quality is often assessed by conventional hole quality measurement systems, such as a coordinate measuring machine, a roundness machine, and a surface profilometer, all of which are widely used for metals [29,30]. Since most conventional hole quality assessment techniques are developed for metallic holes, it is not well understood how the unique CFRP hole damage and fiber pull-out influence hole quality parameters. Through drilling experiments of carbon fiber-reinforced plastic composites, this study investigates the impact of varying drilling feed and speed conditions on fiber pull-out geometries and the resulting hole quality parameters.

## 2. Experimental Procedures

### 2.1. Drilling Experiments

**Figure 2:**
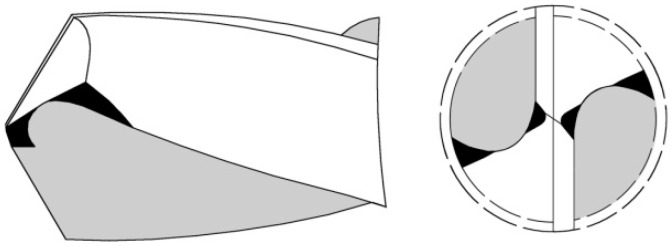
Schematics of the drill and its geometric information.

Experiments were conducted to be a close representation of aircraft final assembly procedures. The CFRP laminates used in this study were aerospace grades, provided by the Boeing Company (Seattle, WA, USA) in the form of plate coupons with a length of 190.5 mm and a width of 152.4 mm. The CFRP workpiece was quasi-isotropic comprised of 40 layers of carbon fiber in epoxy matrix, having a thickness of 7.54 mm. The CFRP layups are [(0/45/90/-45)_4_/90/0_2_/$\bar{0}$]_s_ and the fiber diameter is approximately 5 µm. The drill used in this experiment was a four-facet tungsten-cobalt (WC/Co) drill with the diameter of 9.525 mm and a point angle of 118°. Figure 2 presents the schematics of the drill and its geometric information.

**Figure 3:**
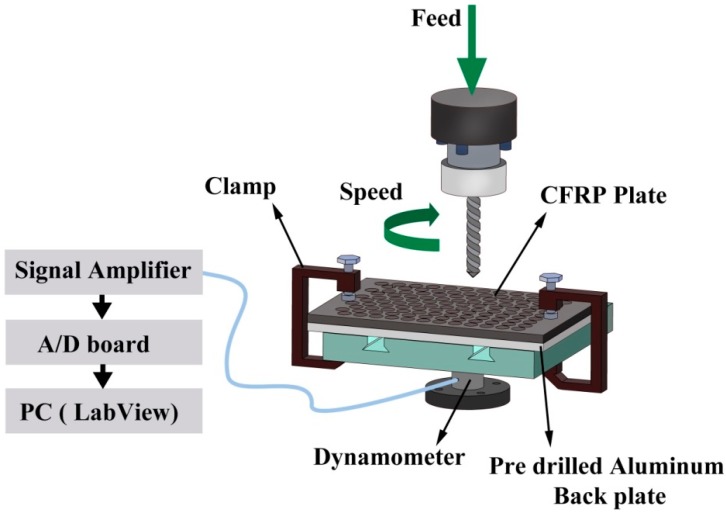
Schematic of the dry-drilling experimental set up.

The dry-drilling experiments were performed using a vertical CNC milling machine (MiniMill, HAAS, Oxnard, CA, USA) with 7.5 horsepower. In order to mitigate the deflection and exit delamination of the CFRP workpiece, a pre-drilled aluminum fixture (hole diameter = 12.7 mm) was utilized in the experiments as a back plate. Figure 3 illustrates the schematic of the dry-drilling set up.

The drilling experiments were conducted with two input variables in the central composite design as shown in Table 1. The output responses include the drilling force, torque, and the machined hole quality parameters, including hole size, roundness, and surface roughness.

### 2.2. Fiber Pull-Out Geometry Assessment

In order to assess the length and depth of the fiber pull-outs on the CFRP-machined holes, scanning electron microscope (SEM) images of the inner surface of the holes were captured. The drilled CFRP holes from hole 16 to hole 24 were cut in half for the assessment using an SEM (Aspex, Delmont, PA, USA), and fiber pull-outs were observed in the entire half holes. The SEM images are used to measure the length of individual fiber pull-out. The detailed method of quantifying fiber pull-out length can be found in Appendix A. 

**Figure 4:**
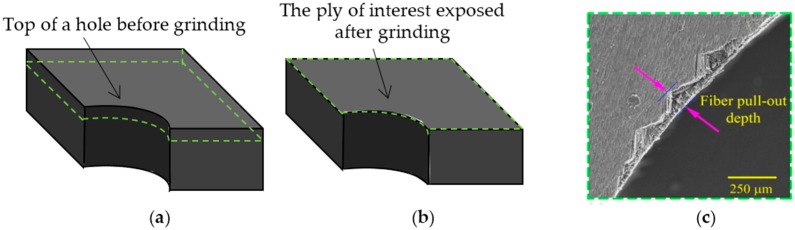
Destructive method of the fiber pull-out depth measurement process; (**a**) as-machined CFRP hole (the dotted line indicate the ply of interest); (**b**) a ground CFRP hole after grinding; and (**c**) a top view of the fiber pull-outs on the ground and polished CFRP hole observed in SEM.

The depth of individual fiber pull-out was assessed by mechanically sectioning the CFRP holes. In this method the cross-section of the hole, as it is shown in Figure 4a,b, was ground and polished from the hole entrance side, down to the ply which contains thefiber pull-outs. The CFRP hole was polished with submicron alumina powders and then placed into the SEM to capture the images of the fiber pull-outs from the top view. Figure 4c shows an example of fiber pull-outs in a SEM image of a sectioned hole. The CFRP top ply removal process was continued for the top eight layers of each hole and each individual fiber pull-out site was analyzed under the SEM in order to measure the maximum depth of the fiber pull-out.

### 2.3. Hole Quality Assessment

The CFRP hole quality parameters chosen for this study are hole diameter, roundness, and surface roughness. Exit hole quality parameters, such as delamination length and the area of protruded fibers, are not assessed due to the use of a back plate on the exit side. This is a standard drilling setup in aerospace manufacturing to minimize exit hole damage. 

In order to measure the hole quality parameters chosen, three measurement devices were used; a coordinate measuring machine, a precision-roundness machine, and a surface profilometer. The coordinate measuring machine, or CMM (Gage 2000 CMM, Brown and Sharpe, Surrey, UK), with a 1 mm diameter ruby spherical tip was manually used to assess the average hole diameter. Each hole’s diameter was averaged from the diameter data at five different levels of an entire hole. At each level, at least 10 points were recorded to create an average hole diameter value. A roundness, or circularity, criterion specifies a tolerance zone bounded by two concentric circles, within which each circular element of the surface must lie and applies independently at any plane. A precision-roundness machine (Talyrond 265, Taylor Hobson, West Chicago, IL, USA) with a 1 mm diameter spherical tip utilizes a rotating gauge head mounted on a vertical column to measure roundness of the hole. With the precision-roundness machine, the spindle is mounted vertically on a stand and the machined hole is mounted on a table placed just below the spindle. The hole is centered by means of the table such that the axis of the spindle coincides with the axis of the hole. The spindle is rotated at 6 rpm from the top to the bottom of the hole with a step size of 0.09 mm, which is half of the CFRP ply thickness. Indicator reading values are recorded for all of the positions. The hole surface roughness was evaluated using a surface profilometer (Surftest SJ-210, Mitutoyo, Kawasaki, Japan) with a stylus tip with 5 µm radius and a 90° taper angle. A cut-off length of 0.8 mm and traveling length of 4.8 mm were used during surface roughness measurements. Arithmetical mean deviation of the roughness profile (Ra) and the maximum height of the roughness profile (Rt) parameters were used to evaluate the surface roughness of the holes. For each hole, nine measurements at 0°, −45°, and +45° were performed to calculate the average roughness.

## 3. Results and Discussion

### 3.1. Drilling Forces

**Figure 5:**
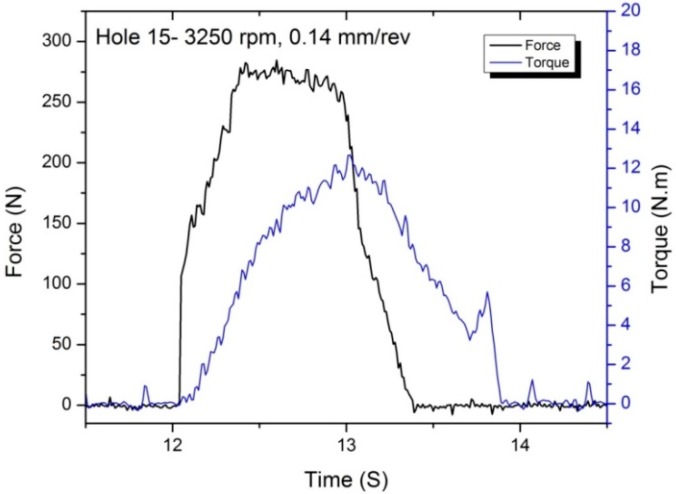
Thrust and torque profiles of drilling a hole at 3250 rpm and 0.14 mm/rev.

**Figure 6:**
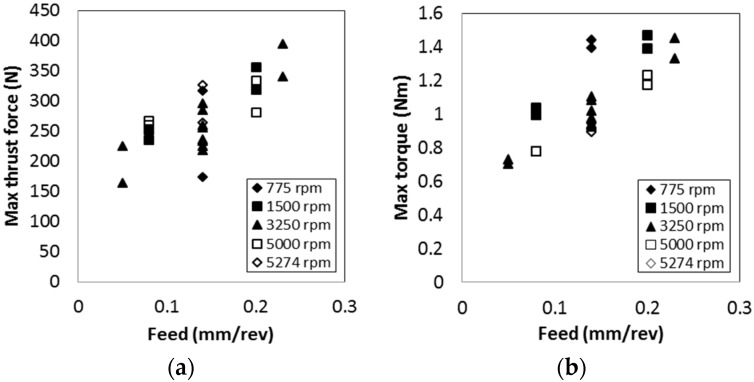
The effect of spindle speeds and feeds on maximum (**a**) thrust force and (**b**) torque.

The thrust force and torque profiles for the hole drilled at 3250 rpm and 0.14 mm/rev are shown in Figure 5. The thrust force suddenly rises when the drill chisel edge makes contact with the workpiece, however, the torque is close to zero. As the cutting lips are engaged through the workpiece, both thrust force and torque gradually increase. Thrust force reaches its maximum at the full engagement of the drill chisel edge and the cutting lips. When the drill chisel edge exits the workpiece, the thrust force starts to decrease rapidly and it goes to zero as the cutting lips cut the remaining workpiece. Unlike the thrust force, torque increases after the full engagement of the drill cutting lips and the maximum torque occurs when the drill tip hits the bottom of the plate as it is about to exit the plate. A small amount of torque remains as the drill exits and retracts; this is due to the engagement of the drill margin and helix with the uncut fibers of the hole wall. Torque reaches zero once the drill completely retracts from the hole. In order to investigate the effect of drilling conditions on the drilling forces, the maximum thrust force and torque values of all of the conditions are plotted in Figure 6.

Both maximum thrust force and torque values increase as the feed increases. This is because, at high feed rates, the undeformed chip thickness under the drill is largely translating into a higher material removal rate and, thus, a higher thrust force. Higher spindle speed resulted in slightly higher thrust force at the constant feed up to 0.14 mm/rev. At 0.20 mm/rev, maximum thrust forces at 5000 rpm are slightly lower than those at 1500 rpm. This may be due to the matrix softening at the high-speed condition. Maximum torque increases by decreasing the spindle speed condition. This is also due to the matrix softening at the high-speed condition. The increasing spindle speed can raise the cutting temperature and soften CFRP, which eventually results in lower torque.

### 3.2. Characterization of Fiber Pull-outs

**Figure 7:**
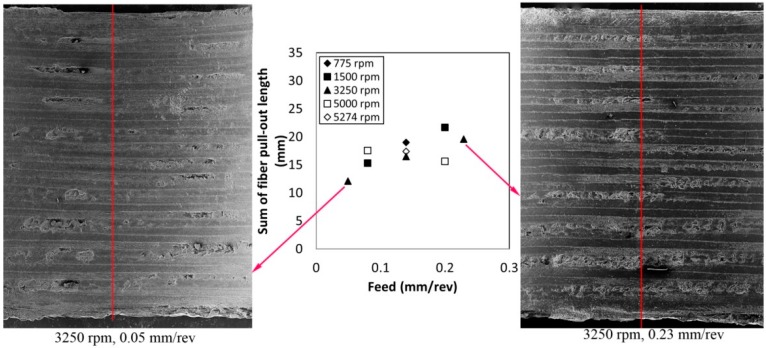
The effect of the drilling conditions on the sum of fiber pull-out lengths with two sample SEM images (the line on the SEM images indicates the center of the half hole surface).

Fiber pull-outs are characterized in two different manners, comprising length and depth. As shown in Figure 7, the total fiber pull-out length of a half hole increases with increased feed. When increasing the feed, the undeformed chip thickness increases, resulting in increasing cutting force. Increased cutting force can extend the range of the angle between the fiber orientation and cutting direction, which ends up increasing the fiber pull-outs in a larger area. For example, the average fiber pull-out length value at the hole from the drilling condition of 0.05 mm/rev and 3250 rpm is 12.1 mm. At the same spindle speed of 3250 rpm, the hole drilled at 0.23 mm/rev has fiber pull-outs with an average length of 19.5 mm. The 0.18 mm/rev difference in feed rate causes an 80% increase of fiber pull-out length on the hole at 0.23 mm/rev when compared with the hole at 0.05 mm/rev. Spindle speed does not seem to have a trend on fiber pull-out length.

**Figure 8:**
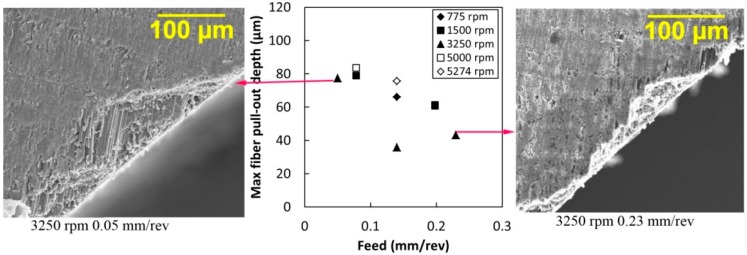
The effect of the drilling conditions on the maximum fiber pull-out depth with two sample SEM images.

The effect of the drilling spindle speed and feed on maximum fiber pull-out depth values is presented in Figure 8. The maximum fiber pull-out depth value decreases with increasing feed. For instance, the maximum fiber pull-out depth for 3250 rpm and a feed of 0.05 mm/rev was 78 µm deep. At the same speed of 3250 rpm and a higher feed of 0.23 mm/rev the fiber pull-out was 41 µm deep. Although the opposite trend has been found in other studies, such as one conducted by Kim et al. [16], the change in fiber pull-out could be related to the tool geometry used. Similar to the results with the fiber pull-out length, the effect of speed on the maximum fiber pull-out depth is inconclusive.

### 3.3. Hole Size

**Figure 9:**
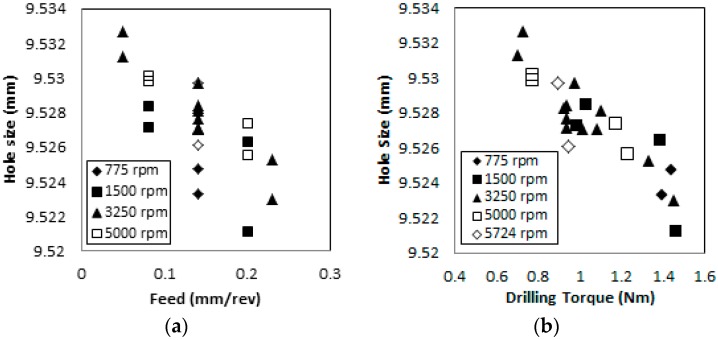
Effects of (**a**) feed and (**b**) maximum torque on the average hole size (drill diameter = 9.525 mm).

Hole size assessment results are shown in Figure 9a. The average hole size decreases as the feed increases. The average hole size reduces by approximately 4.7 µm per feed increment of 0.1 mm/rev. When considering the drill diameter of 9.525 mm, the holes produced higher than 0.15 mm/rev of feed tend to be undersized. The role of feed on the average hole size can be related to the undeformed chip thickness and the cutting force during the process [16,31]. Increasing feed results in increased undeformed chip thickness, which requires the increased cutting force. With increased cutting force, CFRP is pushed downward with the tool and deformed slightly towards the drill margin. After the drill retracts, CFRP springs back, resulting in a smaller hole size [16]. Figure 9a also shows the effect of the drilling spindle speed on the average hole size. Increasing speed entails an increase in the average hole size. This can be attributed to the same phenomenon of thermal expansion of the tool at higher speeds. The trend of hole size variations from various drilling conditions agrees with the past study results done when drilling of glass fiber reinforced plastic composites (GFRP) [32].

Figure 9b shows that the average hole size decreases with increasing maximum torque. Another potential reason for larger holes at lower feeds may be related to the cutting temperature. When the feed is low, the tool engagement time increases. Due to the low thermal conductivity of CFRP, the cutting temperature increases with lower feed conditions. The high cutting temperature with a longer tool engagement time results in tool and workpiece expansion. However, the tungsten carbide tool has thermal expansion coefficient about three times higher as compared with carbon/epoxy composites, which will result in the expansion of the tool and oversized holes in CFRP at lower feeds. As a note, the generated heat when drilling CFRP raised the tool temperature up to 370 °C and resulted in an increase of the hole size as the drilling process is prolonged [33], whereas the generated heat is minimal at higher feeds with the shorter tool engagement time, which results in CFRP hole sizes closer to the drill nominal size. 

The contribution of fiber pull-outs on the hole size assessment results obtained using the CMM are unclear. The CMM tip diameter used in this study is 1 mm, as a result the maximum possible engagement of the tip on the fiber pull-outs being 4 µm when assuming the fiber pull-out width is the same as the ply thickness, or 180 µm. As shown in Figure 8, the maximum fiber pull-out depth value reduced as the feed increased (approximately 20.0 µm per feed increment of 0.1 mm/rev). This is higher than 4 µm, which is the capability of the CMM tip detecting fiber pull-outs. Therefore, the CFRP hole assessment data using the CMM tip with 1 mm diameter may not be accurately influenced by fiber pull-outs. 

### 3.4. Hole Roundness

Table 2 summarizes the roundness analysis results assessed with the CMM. The average roundness values range from 4.978 µm to 6.909 µm. The effect of the drilling conditions is inconclusive with the average roundness. At the constant speed of 3250 rpm, increasing the drilling feed from 0.05 to 0.23 mm/rev increases the roundness error by approximately 0.5 µm. The reason resides in the fact that increasing feed elevates the axial force, which consequently results in higher radial forces that are mainly responsible for drill deformation and poor roundness results [34]. The same trend was found by other researchers while drilling fiber-reinforced plastic (FRP) composites [16,35]. When the average roundness values are compared with the fiber pull-out length and depth data, there seems to be no correlation among them.

**Figure 10:**
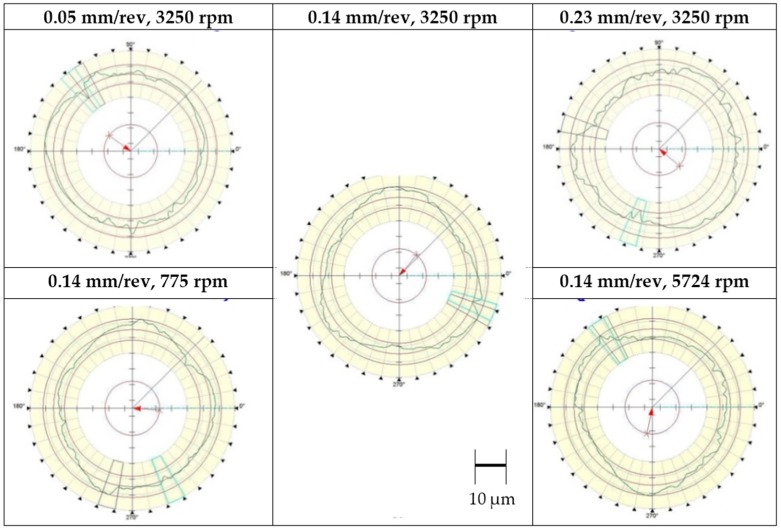
Hole roundness profiles in the middle of the holes.

As shown in Figure 10, the roundness profiles at relatively high feed and high speed contains occasional deep valleys. This matches quite well with the fiber pull-out contour length introduced with the SEM pictures in Figure 7. The precision-roundness machine with a 1 mm diameter tip can capture the fiber pull-outs up to approximately 4 µm. Therefore, the location of fiber pull-outs can be detected by the precision-roundness machine while the fiber pull-out depth cannot be assessed effectively.

### 3.5. Hole Surface Roughness

Results of the surface roughness are averaged and presented in Table 3. The surface quality degraded while the feed rate in mm/min, the product of feed and speed, of the machining conditions is increased. In other words, the higher the feed and speed, the rougher the surface of the holes. However, this increase in the surface roughness value is not unexpected. The same results were found by Khashababa et al. [36] while drilling woven CFRP plates. The non-homogeneous structure of the composites make it difficult to get a uniform surface throughout the hole from entrance to exit. The surface quality in composites is highly influenced by fiber direction during the cutting process. Ramulu et al. [19] found that fibers with orientations above 90° (−45 in this case) are more likely to be pulled out while cutting. Different behaviors of fibers, depending on their directions, sometimes results in inconclusive or insignificant results for surface roughness. Average Ra and Rt values seem to be related more with fiber pull-out length than the fiber pull-out depth. The maximum fiber pull-out depth observed with the destructive method range 38–85 µm, which are far larger than the range of average Rt values. However, the fiber pull-out length increases with an increase in the spindle speed. This means the profilometer stylus may detect fiber pull-outs more often on the holes with the high-speed conditions (5000 rpm or 5724 rpm) at consistent feed rates. It can be concluded that the surface profilometer may be capable of assessing the area of fiber pull-outs, while it may not be able to detect the fiber pull-out depth accurately. 

## 4. Conclusions

The drilling experiment is conducted on quasi-isotropic CFRP plates using a tungsten carbide drill in the dry drilling condition, and following conclusions are drawn:

(1) Both maximum thrust force and torque values increase as the feed increases due to the dependency of the drilling feed to the undeformed chip thickness. Due to matrix softening, maximum torque increases by decreasing the spindle speed condition.
(2) An increase in the feed increased the fiber pull-out length. The maximum fiber pull-out depth, depending also on the drilling feed condition, ranged from 38–85 µm.
(3) The hole size at lower feeds and higher speeds was found to be larger than the nominal drill diameter, which may be due to the instability and thermal expansion of the tool and the workpiece. Roundness of the holes on CFRP plates was found to be influenced by the feed rate change, rather than speed, which is a result of higher radial force in the feed increments.
(4) The effect of fiber pull-outs is minimal to the hole size and roundness assessment results by the CMM. However, the precision-roundness machine is able to detect the fiber pull-out locations of a hole.
(5) The surface roughness results as a factor of the feed rate during the process, increasing the roughness with both cutting speed and feed increments. Surface roughness parameter values measured by the surface profilometer are dependent more on the fiber pull-out length than the fiber pull-out depth.

## Figures and Tables

**Table:** 

Diameter (mm)	Point Angle (°)	Helix Angle (°)	Flute Length (mm)
9.525	118	20	73

**Figure A1:**
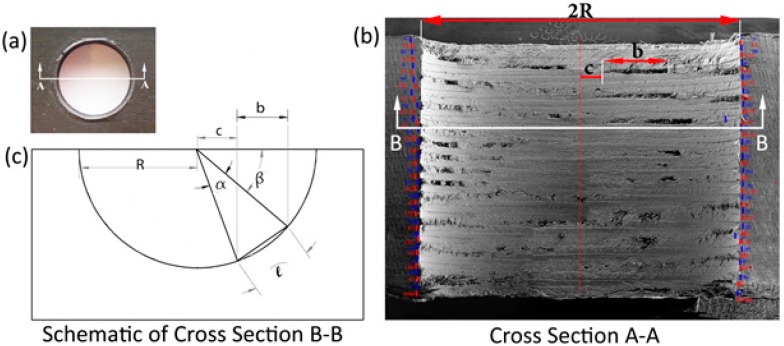
Fiber pull out length measurement technique on cross section of hole. (**a**) Top view-hole entrance; (**b**) SEM image of cross section A–A; (**c**) Schematic of cross section B–B and fiber pull out length measurement method.

**Table 1:** Central composite design for the drilling experiments.

Run Numbers	Spindle Speed (RPM)	Feed (mm/rev)
1, 16	775	0.14
2, 3, 8, 9, 12, 14, 15, 18	3250	0.14
6, 17	5000	0.2
7, 22	3250	0.05
10, 23	5725	0.14
11, 21	1500	0.2
5, 20	5000	0.08
13, 24	3250	0.23
4, 19	1500	0.08

**Table 2:** The effect of the drilling conditions on the average roundness, in µm.

Spindle Speed (RPM)		775	1500	3250	5000	5724
Feed (mm/rev)	0.05			6.375		
	0.08		5.918		5.740	
	0.14	6.350		6.388		6.325
	0.2		5.969		4.978	
	0.23			6.909		

**Table 3:** Effect of the drilling conditions on the average Ra (**top**) and the average Rt (**bottom**), in µm.

Spindle Speed (RPM)		775	1500	3250	5000	5724
Feed (mm/rev)	0.05			2.538 20.985		
	0.08		1.746		2.160	
			14.49		17.056	
	0.14	2.264		2.462		3.443
		18.819		20.302		26.815
	0.2		2.254		2.943	
			20.190		24.951	
	0.23			2.571		
				22.280		

## Appendix A

Figure A1 highlights the fiber pull-out length assessment method. Figure A1a is a top view of a CFRP hole, while a SEM image of the sectioned entire half hole indicates the cross-section of B–B for further fiber pull-out length analysis in Figure A1b. Figure A1c illustrates the schematic of calculating the curve length of the fiber pull-out based on the SEM image of the machined hole. In this study, the fiber pull-out length can be defined as:

*l* = 0.01745(*R ×**α*) (A1)

where:

*α* Fiber pull-out angle or:
  (A2) $$\alpha =\;\mathrm{ArcCos}\left(\frac{c}{R}\right)-\beta$$
β Fiber pull-out initiation angle or:
  (A3) $$\beta =\mathrm{ArcCos}\left(\frac{b+c}{R}\right)$$
*B* Projected length of fiber pull-out length on image
*C* Projected distance of the fiber pull-out from center of hole
*α* + β Fiber pull-out completion angle
*R* the radius of a hole.

The projected length of each fiber pull-out, which is noted as “*b*” on Figure A1b, and its projected distance from the machined hole center, which is noted as “*c*”, are obtained from the SEM image using a commercially available image processing software (ImageJ ver. 1.48, NIH, Bethesda, MD, USA). Using Equations (A1)–(A3), the fiber pull-out angle (*α*), the fiber pull-out initiation angle (*β*), and the fiber pull-out length are calculated.
